# Some Reflections on Being Good, on not Being Good and on Just Being

**DOI:** 10.1007/s12671-014-0350-y

**Published:** 2014-10-22

**Authors:** Rebecca S. Crane

**Affiliations:** Centre for Mindfulness Research and Practice, School of Psychology, Bangor University, Dean Street Building, Dean Street, Bangor, Gwynedd LL57 1UT UK

Over the last 12 years, through my work at the Centre for Mindfulness Research and Practice (CMRP) at Bangor University, I have been engaged with colleagues (both within CMRP and from other training centres in the UK) in an exploration of how to support the development of a robust professional practice context for our own teaching team and for the growing community of mindfulness-based teachers in the UK. There have been a number of academic outputs from these collaborative engagements (Crane, Kuyken, Hastings, Rothwell, and Williams 2010; Crane and Kuyken 2012; Crane et al. 2012a; Crane et al. 2013; Crane et al. 2014).

These academic writings have been largely presented as an exploration of these issues from the perspective of training organisations and the UK Network for Mindfulness-Based Teacher Trainers (2012). The view from these perspectives leads to the aspiration to support the creation of high standards and clear boundaries for professional practice for trainees, students and practising mindfulness-based teachers in the UK context. These explorations and investigations have also though been nourished and influenced by the view from the inside of my experiences of growing my skills as a mindfulness-based teacher and trainer, by my fluxing perceptions of my own competence and incompetence, by being alongside our trainees as they explore these themes in their practice and teaching and by my connection to my own personal meditation practice which began during the 1980s—before there was significant interest in the professional potential for this work. The learning journey that I have engaged in while undertaking these explorations has thus taken place on a number of levels including the personal. Mindfulness practice reminds us and enables us to consciously work with the continual influence of personal interior experience on our behaviour, actions and choices. This reflective piece is intended to bring to the fore some of this ‘back story’ that has influenced the production of academic outputs.

An example of these subjective/objective dynamics in practice is the process of developing and researching the Mindfulness-Based Interventions: Teaching Assessment Criteria (MBI:TAC) (Crane et al. 2013; Crane et al. 2012b). This process employed a third-person stance in relationship to the object of study—the mindfulness-based teaching process. This was necessary to enable the level of objectivity that is required to reliably assess teaching skills. However, it was clear that in-depth first-person, inside perspectives on and understandings of the teaching process were essential in enabling the development team to capture the subtleties of the teaching process. Although these internal processes are not generally publically disseminated, they are completely integral to the investigative process. The current iteration of the MBI:TAC itself is thus the product of exploring the balance between honouring both the importance of specificity, clarity and objectivity and also the importance of the subjective and personal nature of the material being assessed. Inevitably, balancing these tensions results in compromise. Some level of objectivity is lost in ensuring that the less definable aspects of the teaching process are represented.

I recognise that engaging in examinations of issues such as mindfulness teaching competence are part of a personal endeavour to bridge the paradigms that I inhabit: on the one hand, the investigation paradigm of western scientific methodology, with a focus on measurement, outcome and evaluation, and on the other hand, the mindfulness-based paradigm of non-striving, letting be and engagement with process in the moment. There are tensions inherent in this process. I have been sensitive through this time of the place of these UK developments on competence within the international community of mindfulness-based teachers. I had to deal with my own doubts and fears about the potential scepticism of valued and respected mindfulness teaching colleagues around the world. I found myself wanting to reassure them that the tool I was developing with my local colleagues was not simply a tick box list of how to do mindfulness-based teaching or a reductionist approach to a complex process. I also though wanted to develop a methodology that was robust—that did indeed enable experienced trainers to reliably and validly assess teaching integrity.

I was under no illusion about the difficulty of creating a tool which claims to assess what really happens in mindfulness-based teaching. As I embarked, I did not know where it would take us or whether the endeavour was indeed possible. Would we be able to develop a tool which was practical and usable in training and research contexts, which accurately assessed the construct of mindfulness-based teaching integrity and which could reliably be used by assessors? Is it possible to quantify how well a teacher is doing in delivering a mindfulness-based course? Can relational qualities such as warmth and connection be assessed? What about embodied and experientially felt qualities, such as non-striving and present moment focus? As best I could, I brought to the process an open-minded curiosity as to where this journey would take us. Here, I present some personal reflections on the theme of mindfulness teaching competence.

**Some reflections on being good, on not being good and on just being**

You do not have to be good

You do not have to walk on your knees for a hundred miles through the desert repenting

You only have to let the soft animal of your body love what it loves

Mary Oliver (2004)

Following an exploration of the interconnection of our personal mindfulness meditation practice with our teaching at a teacher training retreat I am co-leading, I invite trainees to call into the room words and phrases that speak to the themes that are emerging. Words arise out of the silence—“trust”, “allowing my body to signal to me what is needed”, “surrendering to the moment”, “letting go of plans and agendas”, “deep listening” … and “we don’t have to be good, but we want to be”. All the words and phrases could have been spoken by any one of us, and this last one triggers a particular ripple of recognition in the room. Right now, after two days of collectively sitting quietly with our own experience in meditation practice, we are all in touch with the possibility that Mary Oliver is pointing to in her poem “Wild geese”—that we do not need to be any better than or different from how we are in this moment to be inherently ok, complete and whole just as we are. And, simultaneously, we all recognise the vulnerability that we each carry that is created by the yearning for and the pull towards some aspiration for how things could be different—for ourselves, for others, for the world.

It is a uniquely human capacity to be able to conceptualise what is desirable and aim towards it. My amazing mind can hold an idea of who and how I am now, alongside an idea of who and how I could be in the future. Mindfulness-based teaching is not random. I know when I am receiving teaching from a skilful teacher—it is a clear and tangible experience. I also know that I want to be a skilful teacher. So, my mind makes a leap. I create an ideal of how I would like to be—a conceptualisation of what I would look like if I was one of these skilful teachers. I then set about trying to become these ideas.

In my early forays into mindfulness teaching, my sense of being a fraud was a part of my process that I tried to suppress—the voice that chided me for my lack of skill, that rated my teaching and that chillingly questioned me —“who do you think you are?” The words of Parker-Palmer in his book “The inner life of the teacher” were a reassuring reminder that I was not alone in this experience: “the constant contradiction between how I experienced myself and how other people viewed me created a painful, sometimes crippling sense of fraudulence” (Palmer 1997). I was familiar with this divide between my outer practice and the inner reality of my experience and had experience of managing a sense of lack, inadequacy and shame. In my work as a teacher, I continued doing all the things I had learned over the years to manage within these contradictions: setting myself the project of gaining knowledge, understanding and skills; planning well (lesson plans with by the minute timings, reading notes/books, rehearsing what I would say), showing up with lots of time in hand, getting all the practicalities in place… and while teaching to put a mask in place and keep the sense of something lacking at bay. Once the teaching was over, the sense of fraud would emerge with the full ferociousness and cruelty of post session ruminations.

Acknowledging to myself and my co-teachers that I had waves of feeling like a fraud was an extraordinary first step in discovering that there is another way to sit in the midst of this. In conversation with colleagues, I discovered that this phenomenon was a force for many. It became something I and we could be humorous about and mutually acknowledge the pain of it both during and after teaching.

Gradually, I came to sense the judgements that accused me of being an imposter and a fraud in a different way. I began to recognise them as an aspect of my humanity—to see how common it is for us to find ourselves wanting, how painful it is, how so many of the participants who come to our classes experience this, how many of the trainees who come to our trainings experience this—and how impossible it is for me to connect to fellow humans if I am out of contact with the complete range of experience that makes up my own humanity.

I could see how this sense of gap and discrepancy between the person I found myself to be and the person I was striving to be created strain and tension, and left me sitting with deep shame for being the wrong person. In these moments when my thoughts homed in on this gap, my attentional capacity was so consumed by my conceptual processes that I was cut off from connection with my essential nature. I was out of touch with my direct experience. The feelings that emerged were so painful and so uncomfortable that every cell in my being urged me to push them away, to not acknowledge them, to get beyond them—and then things would be ok. In my efforts to reach beyond the immediacy of my experience I was trying to protect myself from the feeling of vulnerability that these anxieties triggered. I was trying (in vain) to be in control of how I felt.

The root meaning of the word vulnerable is “capable of being wounded or hurt” (Dictionary.com, 2014). My ancestors were very good at protecting themselves from threats to their vulnerability. I have thus inherited a gene pool that wires me to be vigilant to threat, to protect myself from danger. In truth, however, I have never while I am teaching a mindfulness-based class been faced with physical danger. My threat system has instead though been frequently alerted to danger by my own interior experience—my thoughts, sensations and emotions and the action tendencies these illicit in me. My fear that I might mess up is a threat. It arrives as an interconnected cluster of sensations, thoughts, emotions and behaviours which in microseconds can feed cyclical loops of escalating anxiety (see Table 1).

**Table 1:** Elements of experience during a stress moment

Sensations	Emotions	Thoughts	Behaviour/impulses
Hollow sensation in abdomen, Heart pounding fast and loud, Heat rising through trunk, Cold extremities	Fear, Anxiety, Sense of lack and inadequacy, Shame	“Don’t mess up” “Get it right” Rehearsing what to say Self-management inner talk (“just get through, you’ll be ok” etc.)	Tendency to speed up, to listen less well to participants, to move into didactic ‘teacher’ mode, to become time and task focused

I began to get curious. If this is an expression of what it is to be human—can I allow myself to be human? Is it possible to sit in the midst of even this? Can I learn to live in accord with the way things are even in this moment of heart pounding, heat rising anxiety? Is it possible to relax back into being human—to see that ultimately, I am in control of very little? Can I invite my instinctual reptile-like being that is trying to keep me safe by alerting me to danger, to recognise these internal stirrings as passing phenomena rather than threats? Can I allow my being to sit inside this experience of vulnerability, rather than riding the instinctive drive to get away from it by avoiding contact with the “sensory” feel of it and allowing my thinking mind to get to work on problem solving it away?

When I allow it, my personal mindfulness meditation practice offers me a way of hanging out with these experiences from the inside out (as contrasted with the experience of imagining how I might be perceived from the outside in). Sitting in the midst of feeling lost, confused and inadequate, I glimpse that these are not problems to be solved. They are painful *and* bearable. They are inherently human. Not only are they workable in this moment (workable in the sense that I can allow them to be here)—but also they are an asset to my teaching. They keep me directly connected to the truth of human experience. How can I teach if I am not also engaged in an exploration of how to sit in the midst of my life? …an exploration of the ease that can be experienced *within* the midst of moments of heart pounding, heat rising anxiety while we teach a mindfulness-based class or engage in any other aspect of our lives.

Interestingly over time, I notice that the label “anxiety” for this cluster of sensations is a habit in itself. The label is strongly associated with unwanted, feared, deeply unpleasant experience. These associations come along with memories of past experiences of shame and anxiety, and I become entangled with an idea about “my” anxiety. The memories themselves arrive as clusters of images, thoughts, emotions and sensations. Interestingly, de-coupling the sensations in the moment from the label seems to free me from past associations. As I take my seat as a teacher at my most recent teaching event, I sense the arousal in my body as a tapestry of interconnected thoughts, emotions and sensations. Instead of contraction associated with a fear response, I notice a whole mixture of experiences in the moment and seem easy about allowing them to be all in the mix—a joyous sense of aliveness; curiosity, interest, excitement and fear about what will unfold in the space we are creating together; and connection to and compassion for myself and this group of people who I am about to journey with.

When I cast my mind back over the years to the teachers who have affected me and whose teachings I have connected with, I discover that they are people who have revealed their “personhood” to me. I can tangibly feel both the vulnerability and strength of their humanness as they teach. They are embodying a possibility—a way of carrying the whole of themselves into each moment rather than partitioning unwanted aspects of their being away from the moment. My participants are not looking for me to be perfect. They are looking for a human being who is in touch with and sensitive to the realities and consequences of being human—someone who has an expanded capacity to bear the imperfect and the irreconcilable, someone who has a willingness to hold paradox without trying to solve it. Mindfulness invites me to be with my imperfections and ironically it is *through* this that I am able to connect with my participants. Any didactic teaching that I have to share with my participants would be abstract ideas if it is not integrated with an embodied sense of the possibility that is being explored.

A curious sense of competence in the context of mindfulness-based teaching emerges. It is very different from the ideas of competence that I have grown up with. I have spent many years valuing and training the capacity to know what is needed and when it is needed, to get things done quickly and easily, to meet targets and objectives, to follow a clear agenda, to be efficient and on top of things, to have clarity of purpose, not to doubt, and to develop expertise. In Mary Oliver’s words, I have learnt how to “be good” (2004). These capacities serve me well in many aspects of my life. However, when I apply them to managing my interior personal world, I make my emotions, thoughts and sensations into problems that I need to solve, manage and overcome. I am learning through my mindfulness training to expand into a whole new area of “competencies”. These include being attuned to my own and other’s vulnerability, being willing not to know where each moment will take us but also being open to whoever I am moment by moment and to whatever is emerging, giving time to things so that there is space to sense the body, loosening up on my personal identification with experience, having clarity of intention while being non-striving in the moment, riding lightly with the waves of experience that come along with being human and living honestly and authentically with myself by working from the truth of experience rather than some idea of how it could or should or might be.

So, the paradox is that in the context of mindfulness-based teaching, the competence we are aiming for is to be able to be completely and authentically the person we *already* are, to allow myself to be the person I am moment by moment and to let go of the expectations that I or others may have for me (and mostly they are imposed from within!). I experience huge relief when I let go of striving to make the teaching and myself into “something” and instead place priority on connection. Discovering how it is to sit within the creative tension of holding an aspiration for myself and my participants, while putting all my attention into the process and allowing things to be completely as they are and complete as they are. Sensing into discovering how this moment is expressing itself and using connection with the immediacy of my own experience to support me in attuning to the wider process of the participants and the teaching.

Somehow, in developing the ability to be with our own experience, we are opening up our capacity to be touched by it and by the experience of others and to connect with others by our way of being. Clearly, there are some things that we need to know and understand to enable us to become skilled mindfulness-based teachers, but at the heart of mindfulness teaching is an exploration of what it is to be human. We are not serving the teaching if we strive to push through and beyond our human vulnerability in order to show up in the classroom. As Parker-Palmer writes “we cannot see the fear in our students until we see the fear in ourselves” (Palmer 1997, p.27).

Dauntingly, as I journey further into my exploration of what it is to teach mindfulness-based classes, I discover that I have taken on the biggest challenge that my life can present to me—one that takes me right to the core of my basic vulnerability. The tool for this “job” of teaching is to be the person I am. I am the vehicle for conveying the teaching. Through me putting my being into the teaching process, participants have the opportunity to taste and experiment with a new possibility within themselves. This same theme is expressed by a trainee at the end of a teacher training retreat:

Trainee: “I have never felt so much fear while teaching”

Me: “say some more, I’m really interested…”

Trainee: “You asked us to be authentic while teaching – that was a huge challenge. I am a really experienced teacher – I know how to stand in front of a high powered audience and deliver a talk on stress. I can do that. But to do this whilst also being directly in touch with my own experience and allowing this to be a part of the process was new. It felt less safe. I felt less in control and more anxious - but I did feel like I was in connection with myself and the group I was teaching. The feedback I received was extraordinary – my teaching had touched them in some way.”

The connection that the trainee describes with her own experience and with the group she was leading elicits a ripple of recognition in me. When I can truly be at ease with myself even in the midst of anxiety, I naturally feel a deep sense of connection with my participants. In these moments of connection, I am in touch with my vulnerability but not in a way that elicits ruminative self-doubt. I am connected in ways that supports my capacity to compassionately be present with experience.

So, what is the connection between the outer “view” of what looks and feels like good teaching and what it feels like from the inside? From the outside, good teaching does consist of the MBI:TAC domains. However, my inner experience of teaching has little awareness of or concern with those domains. If my attention was placed on them, I would be out of connection with immediacy and into connection with the realm of concepts and ideas. Just as Dreyfus and Dreyfus (1986) describe in their work on competence, there is though a natural movement through early phases in which we do need to give some of our attentional capacity to the “ideas about” the skill as we gradually assimilate and integrate the craft into our being, to later phases in which the process becomes intuitive and fluid.

The particular curiosity in this context is that the instrument for this craft of teaching mindfulness is ourselves. Many of the competencies that we are developing are deeply personal—understandings about and a way of holding myself and the world. The way we tune the instrument and develop our skills to draw out the best from this instrument is to bring mindful attention to our experience both in everyday life and during formal meditation practice. The patterns that emerge in my meditation and teaching practice are no different to those that emerge in the rest of my life—working too hard, wanting some things, not wanting others, wanting to be liked, sensing myself as separate, wanting to be good, fearing being bad. Much of this learning seems to be a process of “undoing” which is very different from my well-learned patterns of accumulating knowledge and understanding—a continued movement from the cognitive, learn-how-to-do-it and try-to-get-it-right place to trusting my innate, inner wisdom and presence in the moment. This is not work that I can accomplish and then move on from—it is a lifelong journey. Who the real, authentic “me” is, is a mystery—a constant on-going discovery of who I am in this moment and of how my life is expressing itself now. This is true for the whole of my life yet my meditation practice and my practice as a mindfulness teacher goes straight to it by stripping away the layers of other stuff that often seem to be the explicit thing that is going on. The implicit becomes explicit.

After I write this piece, I realise that I have no way of evaluating it. It is simply an expression of my experience over the years. Soon after when I walk out of teaching a mindfulness class, I meet a colleague in the corridor. “How did your class go?” I say “fine, it was good”—but realise as I am saying it and later when I reflect that this was an automatic response. In truth, I don’t know. I can’t evaluate it. I was in touch with my experience (moments of sensing: contact of body with chair, fluxing sensations in my abdomen, contracting, opening…emotions arising: warmth, connection with the group and individuals, disconnection, confusion, sadness, joy…thoughts moving through…) but somehow it is not easy to gather all this together into an honest answer to the question: “how did your class go?” It doesn’t seem amenable to being good or bad. I am left very curious about the interface between my experience of the immediacy of my experience and my teaching competence in the context within which I practice. Just now as I sit and write I feel very aware of the tender vulnerability that resides at this interface.

Beyond our ideas of right-doing and wrong-doing, there is a field. I'll meet you there. When the soul lies down in that grass, the world is too full to talk about. Ideas, language, even the phrase 'each other' doesn't make sense any more.

Jelaluddin Rumi (1995)

I also have a heightened awareness that at the core of my work is a personal aspiration to build bridges and to support conversation between the paradigms that I find myself inhabiting: on the one hand, the paradigm of mindfulness with its emphasis on building and sustaining connection to the immediacy of experience and its ethical framework which includes deep care and concern for life and the interconnected web that nourishes it and on the other, the paradigm that predominates in mainstream institutions of striving for excellence, judging, quantifying, measuring, assessing, problem solving and achieving.

Mindfulness-based teaching is based on the premise that in order to live a balanced and meaningful life, we need (on a personal level) to be able to intentionally move between our doing mode of mind and our being mode of mind. Exploring this balance point and the way to integrate being and doing is a core part of my personal practice. It is also at the centre of my work in the world. My work is sustained by an aspiration that there is potential for greater balance within our institutions and ultimately within the structures that make up our society.
